# Extending the breadth of African laboratory medicine

**DOI:** 10.4102/ajlm.v8i1.1128

**Published:** 2019-12-05

**Authors:** Iruka N. Okeke

**Affiliations:** 1 Department of Pharmaceutical Microbiology, University of Ibadan, Ibadan, Nigeria

The *African Journal of Laboratory Medicine*’s (AJLM) eighth volume contains almost 30 articles, our largest issue yet. A few areas of laboratory medicine are not represented, but the impressively expansive scope of the volume extends from bacteriology (including mycobacteriology), clinical chemistry, clinical pharmacology, haematology, histopathology and virology to informatics, laboratory development and quality management. While appreciating that growth has come with good subject area representation, we have to be just as intentionally concerned with the diversity of matters other than content.

If nothing is done, some estimates suggest that it will be over 200 years before gender parity is reached throughout society^1^ and other forms of imbalance may be even more entrenched. AJLM is therefore not alone in its current introspection and quest for diversity. In a bid to accelerate the quality improvements that will come with inclusiveness, a number of editors, journals and independent researchers have published their recent observations and reflections on the gender and geographic balance of editors, reviewers and authors.^2,3,4^ Almost universally, they find that representation in scientific journals is slanted towards the male northern bias that permeates most of science, even if the tilt angles differ.

As a journal owned by an African scientific society, with both editorial and publishing offices based on the continent, AJLM has an uncommon position in this discourse. Firstly, women are severely underrepresented in African science^5^ and globally women play a prominent role in healthcare delivery but are much less visible in its leadership.^6^ Both of these deficits could well influence representation in a laboratory medicine journal with a lens on Africa, the continent that is home to the fewest scientists per capita. Secondly, as a regional journal with global reach, we receive and publish a large number of scientific articles from African authors. We simultaneously provide an owned perspective and a global view of African science, a dual role that is rare in scientific publishing.^2,4,7^ This vantage-point notwithstanding, AJLM also represents a powerful mouthpiece for speaking to African scientists and therefore we cannot take for granted that our editorial processes and authorship will reflect our readership.

The vision and content of a journal is shaped by its editors. As of October 2019, AJLM’s editorial team comprised a female editor-in-chief, five section editors (two female) and a 22-person editorial board that included only three women. This team had affiliations on four continents with a third of us working in Africa. With three of our six editors being women (not counting our managing editor, who is also female), we merit only 60% by Bhaumik and Jagnoor’s^8^ Composite Editorial Board Diversity Score. But, given our focus on Africa, we have a greater proportion of Africa-based editors than any of the 27 global health journals that were assessed in that study. We lag most noticeably in geographic diversity. Scientists from all over the world work with laboratory medicine experts on our continent to better health, and findings from studies in or on Africa will inform the practice of laboratory medicine anywhere. So, our editorial board is deliberately global even though we do seek editors who are familiar with science in Africa. We, therefore, would benefit from representation from Australasia, Latin America and the Caribbean, which is currently absent from our editorial board.

Even though this is not the case with respect to African biomedical research overall,^5^ the vast majority of our articles are wholly or predominantly authored by Africans. In spite of the well-documented male skew in African science,^5^ AJLM Vol. 7(1) 2018 comprised articles with nine female and five male first authors (with one indeterminate). First authors on articles in Vol. 8 include 11 men and nine women (4 indeterminate based on first name and online searches). Therefore, AJLM is an important venue for publishing female-led work, particularly from Africa. We are also pleased to find that AJLM continues to feature articles from a range of African countries and from beyond them and that it addresses health issues of different populations, including ethnic minorities or those living in rural or remote areas.

Unfortunately, while we have good representation from Africa, we find that there is less diversity in the geographic origin of AJLM articles within the continent. Five 2018 corresponding authors listed South African addresses and one each from Rwanda, Ethiopia, the Netherlands, Kenya, Cameroon, Barbados, Haiti, Namibia, Nigeria and Canada. In 2019, we published six articles with corresponding authors from South Africa, four each from Nigeria and the United States (US), one with a double Nigeria-US affiliation, two from Kenya and Saudi Arabia and one each from Belgium, Botswana, Cameroon, Ethiopia, Ghana and Zimbabwe. While preponderance of Anglophone corresponding authors is expected for an English-language journal, the underrepresentation of submissions from Western, Central, North and East Africa in our recent volumes is obvious and concerning. If AJLM has uncommon success in publishing high-quality African research, it should do so for those parts of the continent least represented in science.

What could AJLM do to enhance diverse representation in our journal going forward? Firstly, we need to maintain the diversity that we do have and to ensure that with the growth in our size and reputation, minority contributors do not become overshadowed or, worse, replaced. AJLM will continue to encourage female scientists to submit to, review for and edit for the journal as well as to support those that work with us. We will also continue to offer a mentored review process and copy-editing support for accepted articles, which allows us to feature work by new authors and non-Anglophone scientists. However, we will have to do much more to bring in manuscripts from across the continent and explicitly wish to call for papers from countries not featured in our last two volumes.

From time to time, AJLM offers heavily subscribed manuscript writing training workshops and in future, we will prioritise early-career participants from African countries who are underrepresented in the journal or who are female. We will also endeavour to invite more female scientists to review for the journal and take places on our editorial board. Finally, with this editorial, AJLM is in the process of keeping track of the demographics of our authors, reviewers and board because, unless we do, we will have no idea how far we are from our goal of being the primary outlet for truly balanced scientific work in laboratory medicine across the continent.
